# Multidimensional Analysis of a Cell-Free DNA Whole Methylome Sequencing Assay for Early Detection of Gastric Cancer: Protocol for an Observational Case-Control Study

**DOI:** 10.2196/48247

**Published:** 2023-09-20

**Authors:** Yongjun Han, Jiangpeng Wei, Weidong Wang, Ruiqi Gao, Ning Shen, Xiaofeng Song, Yang Ni, Yulong Li, Li-Di Xu, Weizhi Chen, Xiaohua Li

**Affiliations:** 1 Department of General Surgery First Hospital of Yulin Yulin China; 2 Department of Gastrointestinal Surgery Xijing Hospital Air Force Military Medical University Xi'an China; 3 Genecast Biotechnology Co, Ltd Wuxi China

**Keywords:** gastric cancer, circulating cell-free DNA, early detection, methylation, fragmentation, chromosomal instability, whole methylome sequencing, multidimensional model

## Abstract

**Background:**

Commonly used noninvasive serological indicators serve as a step before endoscope diagnosis and help identify the high-risk gastric cancer (GC) population. However, they are associated with high false positives and high false negatives. Alternative noninvasive approaches, such as cancer-related features in cell-free DNA (cfDNA) fragments, have been gradually identified and play essential roles in early cancer detection. The integrated analysis of multiple cfDNA features has enhanced detection sensitivity compared to individual features.

**Objective:**

This study aimed to develop and validate an assay based on assessing genomic-scale methylation and fragmentation profiles of plasma cfDNA for early cancer detection, thereby facilitating the early diagnosis of GC. The primary objective is to evaluate the overall specificity and sensitivity of the assay in predicting GC within the entire cohort, and subsequently within each clinical stage of GC. The secondary objective involved investigating the specificity and sensitivity of the assay in combination with possible serological indicators.

**Methods:**

This is an observational case-control study. Blood samples will be prospectively collected before gastroscopy from 180 patients with GC and 180 nonmalignant control subjects (healthy or with benign gastric diseases). Cases and controls will be randomly divided into a training and a testing data set at a ratio of 2:1. Plasma cfDNA will be isolated and extracted, followed by bisulfite-free low-depth whole methylome sequencing. A multidimensional model named Thorough Epigenetic Marker Integration Solution (THEMIS) will be constructed in the training data set. The model includes features such as the methylated fragment ratio, chromosomal aneuploidy of featured fragments, fragment size index, and fragment end motif. The performance of the model in distinguishing between patients with cancer and noncancer controls will then be evaluated in the testing data set. Furthermore, GC-related biomarkers, such as pepsinogen, gastrin-17, and *Helicobacter pylori*, will be measured for each patient, and their predictive accuracy will be assessed both independently and in combination with the THEMIS model

**Results:**

Recruitment began in November 2022 and will be ended in April 2024. As of August 2022,250 patients have been enrolled. The final data analysis is anticipated to be completed by September 2024.

**Conclusions:**

This is the first registered case-control study designed to investigate a stacked ensemble model integrating several cfDNA features generated from a bisulfite-free whole methylome sequencing assay. These features include methylation patterns, fragmentation profiles, and chromosomal copy number changes, with the aim of identifying the GC population. This study will determine whether multidimensional analysis of cfDNA will prove to be an effective strategy for distinguishing patients with GC from nonmalignant individuals within the Chinese population. We anticipate the THEMIS model will complement the standard-of-care screening and aid in identifying high-risk patients for further diagnosis.

**Trial Registration:**

ClinicalTrial.gov NCT05668910; https://www.clinicaltrials.gov/study/NCT05668910

**International Registered Report Identifier (IRRID):**

DERR1-10.2196/48247

## Introduction

Gastric cancer (GC) is one of the most commonly diagnosed digestive tract tumors, accounting for approximately 1.08 million new cases and an estimated 769,000 deaths in 2020 worldwide (7.7% of total cancer-related deaths), ranking fifth for age-standardized incidence and fourth for cancer-related mortality worldwide [1]. China is among the countries with the highest incidence rate of GC, although a gradual decline has been observed in recent years [2,3]. Early GC detection and its prompt treatment are strongly associated with improved prognoses for patients with GC. In Japan and South Korea, population-wide screening has been extensively implemented, resulting in effective diagnosis of tumors at early stages and improved survival rates [4]. Unfortunately, in China, most patients are diagnosed with advanced GC, probably owing to the large population and lack of standardized screening programs. While gastroscopy (upper gastrointestinal endoscope) and biopsy procedures conducted during gastroscopy remain the gold standard methods for GC diagnosis, gastroscopy is an invasive operation often met with poor compliance. Moreover, insufficient endoscopy physicians and endoscopic equipment, as well as the lack of a standardized endoscopic examination in China, present challenges for endoscopy to be used as a population-wide screening method [5].

Currently available noninvasive blood biomarker–based approaches could serve as a step before endoscope diagnosis and aid in identifying the high-risk GC population. However, these methods are associated with high false positives and high false negatives, which can potentially lead to overdiagnosis or underdiagnosis. Serological indicators such as *Helicobacter pylori* antibodies lack specificity for GC detection due to their strong association with atrophic gastritis and gastric ulcers [5]. Other indicators, including serum pepsinogen (PG), serum gastrin-17 (G-17), and MG7-Ag, are not recommended for standalone use due to their poor sensitivity and specificity [6]. A new GC score system based on age, sex, PG, G-17, and *H pylori* antibodies was introduced in China’s early GC screening process expert consensus [5]. While this new GC score system might exhibit improved prediction performance [7], the cost-benefit of its clinical application needs to be analyzed. Plasma tumor markers, such as carcinoembryonic antigen, cancer antigen (CA) 19-9, CA 72-4, CA 125, and CA 242, are linked to patient prognosis but are not suitable for early cancer detection [8]. Furthermore, circulating molecules such as miRNAs have garnered attention; however, their sensitivity and specificity are limited [9,10].

Rapid advancements in technology and analysis have identified cell-free DNA (cfDNA) as a new, simple, and noninvasive method for early cancer detection, offering enhanced specificity and sensitivity. The cancer-related features of cfDNA have been exploited in several aspects to distinguish cancer samples from healthy cases: (1) genetic alterations [11-13], (2) microsatellite instability [14], (3) hypermethylation or hypomethylation of certain genes [15,16] or across the genome [17-19], (4) chromosomal alterations [20], and (5) unique fragmentation patterns [21,22]. Among these aspects, identifying cfDNA genetic alterations poses a challenge due to the low fraction of mutation-bearing circulating tumor DNA (ctDNA) fragments in early-stage diseases or certain tumor types [23,24]. The combination of multiple features has enhanced detection sensitivity in comparison to relying on a single feature, as studied in kidney cancer [25], hepatocellular cancer, and pancreatic ductal cancer [26].

**Figure 1:**
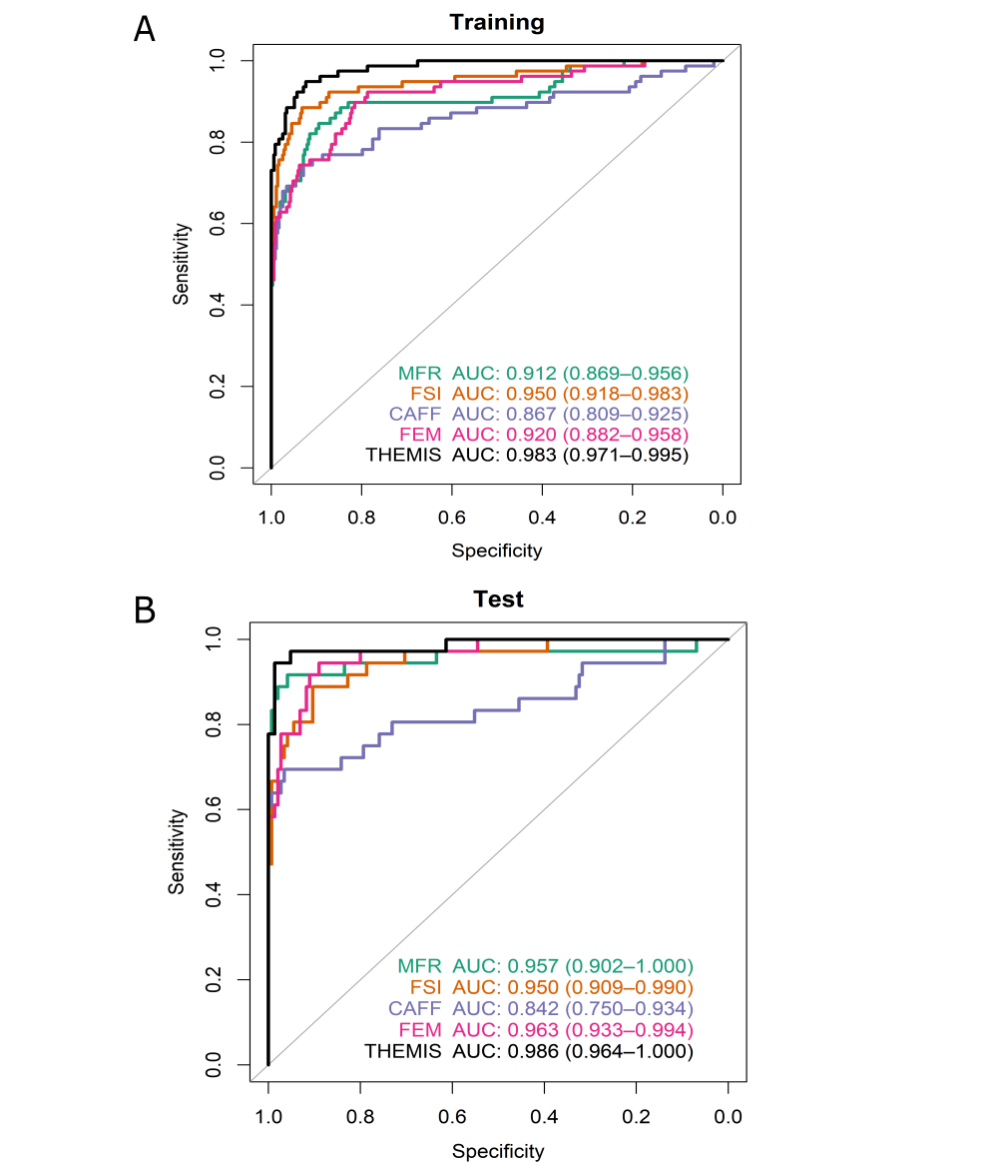
Receiver operating characteristic curve analysis showing the cancer prediction performance of the THEMIS model in the GC subgroup: (A) training data set; (B) test data set. The AUC together with the 95% CI is shown. AUC: area under the curve; CAFF: chromosomal aneuploidy of featured fragments; FEM: fragment end motif; FSI: fragment size index; MFR: methylated fragment ratio; THEMIS: Thorough Epigenetic Marker Integration Solution.

We have also previously developed a multidimensional ensemble model named Thorough Epigenetic Marker Integration Solution (THEMIS). This model integrates various cfDNA features, including methylated fragment ratio (MFR), chromosomal aneuploidy of featured fragments (CAFF), fragment size index (FSI), and fragment end motif (FEM), enabling highly sensitive early detection across a pan-cancer cohort [27]. In a subgroup comprising 114 patients with GC and 497 healthy controls, the THEMIS model showed an estimated area under the curve of 0.983 for the training data set (Figure 1A) and 0.986 for the test data set (Figure 1B). However, the fact that the model was trained on a pan-cancer cohort and the control group did not contain individuals with benign gastric diseases limits its predictive reliability for GC.

**Figure 2:**
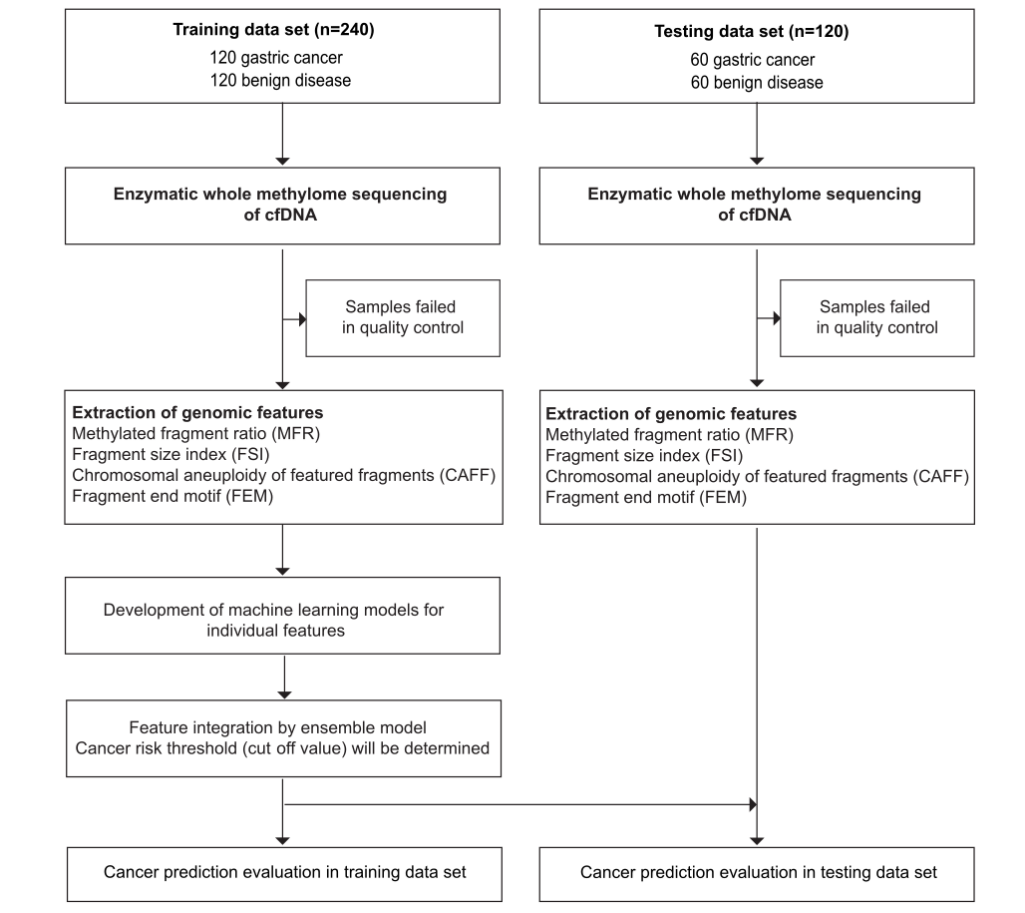
Flowchart of patient recruitment, sample collection, and statistical analysis strategy. cfDNA: cell-free DNA.

This study aimed to develop a highly sensitive GC-specific THEMIS model for the early detection of GC. This study’s design is illustrated in Figure 2. Patients with GC will be enrolled in the malignant group, while the control group will comprise not only healthy individuals but also participants with benign gastric diseases, such as gastritis, gastric ulcers, and gastric polyps. Consequently, a novel model will be developed to differentiate malignant cancer from nonmalignant gastric status. A nonbisulfite enzymatic whole methylome sequencing (WMS) assay [28] will be applied to ensure the preservation of methylation signals from the cfDNA alongside the molecular fragmentation profile. The sequencing data generated from cfDNA will then be analyzed across the abovementioned multiple dimensions, encompassing methylation patterns, chromosomal copy number alterations, and fragmentation profiles. In addition, GC-related biomarkers, such as PG, G-17, and *H pylori*, will be evaluated either independently or in combination with cfDNA features during model training to enhance the detection sensitivity. The overall clinical performance of the model, as well as its performance in each clinical stage, will be evaluated.

## Methods

### Study Objectives

This study aims to develop and validate a novel blood-based WMS method followed by the development of a multidimensional model for the analysis of various features of cfDNA specifically for early GC detection. We expect that this noninvasive liquid biopsy assay will be instrumental in distinguishing individuals with a high probability of GC. Individuals who receive a positive test result will subsequently obtain a confirmed diagnosis by gastroscopy. Conversely, individuals who receive a negative test result will considered as having a low probability of GC. For this group of patients, they would be recommended to undergo a deferred gastroscope, and possibly a repeated test annually.

The primary objective is to validate the overall diagnostic specificity and sensitivity of the model and additionally assess its performance in each clinical stage of GC. The secondary objective is to investigate the specificity and sensitivity of the model when combined with potential biomarkers such as PG, G-17, or *H pylori* levels, aimed at enhancing early GC detection.

### Study Population

This study will enroll individuals who receive gastroscopy at Xijing Hospital of Air Force Military Medical University, Xi'an, China. Further, 2 groups will be formed based on the outcomes of gastroscopy: a malignant group and a nonmalignant group. The malignant group will include patients diagnosed with high-grade intraepithelial neoplasia or GC (more than 90 patients corresponding to >50% of patients in stages I and II), while the nonmalignant group will include both healthy individuals and patients with gastritis, gastric ulcers, gastric polyps, or other benign gastric diseases.

Clinical parameters, such as personal history, family history of digestive tract cancer, gastroscopic findings, and GC staging, will be systematically collected for each subject. Blood samples will be collected for WMS analysis, the results of which will be used to establish a GC diagnostic model.

### Inclusion Criteria

Subjects will be enrolled after understanding and signing the informed consent form, ensuring their eligibility based on the provided inclusion or exclusion criteria.

Inclusion criteria must be fulfilled: (1) age 18 years or older, male or female; (2) availability of complete clinical information; and (3) agreement of the patient to join this study with signed consent and good compliance.

An additional specific inclusion criterion for subjects to be included in the malignant group must be fulfilled: confirmation of gastric adenocarcinoma through histopathology in accordance with the American Joint Committee on Cancer’s 8th Edition Cancer Staging Manual, with a pathological stage ranging from stage I to IV. This includes patients with esophageal gastric junction adenocarcinoma, regardless of histology types.

### Exclusion Criteria

Subjects will be considered ineligible if any of the following exclusion criteria are met: (1) they have previously been diagnosed with any kind of malignant tumor, (2) they have received total or partial gastrectomy, (3) they have received bone marrow or organ or stem cell transplantation, (4) they have received blood transfusion within the past 6 months, (5) they have previously received any form of local or systematic antitumor treatment, (6) they had ongoing fever or received anti-inflammation therapy within 14 days prior to blood draw for this study, (7) if female, they were pregnant or lactating, and (8) clinical information was incomplete or the subject was unqualified to participate in this study.

### Discontinuing Study Interventions and Patient Withdrawal

If the ongoing study is later considered to be infeasible, the principal investigator shall submit a termination application to the institutional review committee (IRC). The decision for the termination will be made by the IRC. Following the decision, the principal investigator shall provide a clinical trial termination report to the IRC.

Subjects may be withdrawn from this study for the following reasons: (1) subjects request to exit the trial and withdraw informed consent, (2) subjects refuse to have blood samples collected, even after providing informed consent, (3) sufficient blood sample collection is difficult for the subjects, and (4) subjects enrolled by mistake and are later assessed to be unsuitable for this study.

### Sample Collection

In total, 20 ml of peripheral blood will be collected from each subject and stored in a Cell-Free DNA Storage Tube (Cwbiotech). Each subject’s information, such as index, name, sample type, and collection time, will be marked clearly. Blood samples shall be delivered to the laboratory within 72 hours at 15 °C-35 °C to proceed with plasma extraction. In situations where timely delivery to the laboratory is not feasible, the blood samples shall be centrifuged at 4000 × g for 15 min at 4 °C and the upper layer containing plasma will be transferred to a new tube. Approximately 4 ml of plasma will be obtained and stored at −80 °C until use. Alternatively, for a period of no more than 1 month before transportation, the plasma can be stored at –20 °C.

### cfDNA Extraction

The plasma samples that were prepared in the previous step will be processed for cfDNA extraction by using the MagMAX Cell-Free DNA Isolation Kit (Thermo Fisher Scientific) per the manufacturer’s instructions. The quantity and quality of extracted cfDNA will be assessed with a Bioanalyzer 2100 (Agilent).

### WMS Library Construction

The entire amount of extracted plasma cfDNA (capped at 15 ng if exceeding what is needed) for each sample will be used to generate WMS libraries with the NEBNext Enzymatic Methyl-seq Kit (New England Biolabs) according to manufacturer instructions, with 1 modification: 100 ng of carrier RNA (TIANGEN) will be added before denaturation by sodium hydroxide. The generated libraries will be amplified with 9 polymerase chain reaction cycles, followed by quantification using the Qubit dsDNA HS Assay Kit (Thermo Fisher Scientific). After that, the constructed libraries will be subjected to sequencing on a NovaSeq 6000 system (Illumina) with a paired-end read length of 100 bp.

### Model Establishment

The estimation data set will be used to establish a machine learning model aimed at predicting cancer probability. Various features associated with plasma cfDNA fragments, such as their methylation status, will be subjected to data preprocessing, feature filtering, and model selection. This will lead to creation of respective classifier models. In a prior context, we defined these features as MFR, CAFF, FSI, and FEM.

#### MFR Feature

Genomic methylation status undergoes substantial alterations even in the early cancer development stage. The release of ctDNA by cancer cells can lead to changes in the methylation levels of cfDNA present in the plasma. To quantify these changes, the whole genome will be tiled into nonoverlapping 1 Mb windows. The methylation levels within each window will be quantified by the fraction of fragments featuring fully methylated CpGs, thereby resulting in the computation of MFR.

#### CAFF Feature

Cancer cells often exhibit chromosomal instability, with instances of partial or complete amplification or loss of chromosomal arms [29,30]. To account for this, a previously described plasma aneuploidy score will be calculated for each sample. This calculation will rely on the levels of copy number alterations in 5 specific chromosome arms, which exhibited the most significant copy number alterations when compared to baseline samples [20].

#### FSI Feature

The size frequency of ctDNA fragments released from cancer cells differs from that of cfDNA [31]. By tiling the whole genome into nonoverlapping 1 Mb windows, the read ratio between short fragments (100-166 bp) to long fragments (169-240 bp) will be computed, resulting in the determination of the FSI. Subsequently, the FSI of each sample will be contrasted with the healthy population baseline, and the cancer risk for each subject will be estimated.

#### FEM Feature

The preferred FEMs of ctDNA differ from those of cfDNA due to DNA nuclease downregulation in the cfDNA fragmentation process [31]. The frequency of a total of 256 4-nucleotide (ie, 4-mer) fragment 5’ end motifs will be analyzed, with the following modifications: (1) only fragments shorter than 171 bp will be selected for analysis, and (2) only reads mapped to the Crick strand will be used for calculation.

An ensemble classifier named THEMIS has been developed based on a generalized linear model incorporating elastic-net penalization. THEMIS is established by integration of the 4 abovementioned features, which includes the predicted cancer probability scores by MFR, FSI, and FEM along with the plasma aneuploidy scores of CAFF. R packages such as *CARET* under 20-fold cross validation will be used.

### Sample Size Estimate and Statistical Analysis

The sample size was estimated using the strategy mentioned by Negida et al [32]. Based on the abovementioned pilot study, the ensemble THEMIS model reached a sensitivity of 0.94 with 0.95 specificity (Figure 1). We expect that the new model integrating similar features as THEMIS will attain a sensitivity of at least 0.9. If we consider the 95% CI width of 0.08 (2-sided test α=.05), then at least 54 cancer cases will be required. If we also expect a certain amount of dropout, such as 10%, a minimum of 60 cases will be needed. The same number of patients will be enrolled in the nonmalignant control group for this case-control study. The plan is to allocate both groups of subjects by the same 2:1 ratio into training and testing data sets. Ensuring at least 60 cases and 60 controls within the testing data set, a total of 360 subjects (180 cancer cases and 180 controls) will be enrolled. This ensures that each group is composed of no fewer than 180 subjects. The malignant group and the control group will be matched by age and sex to minimize possible confounding factors (Figure 2).

The area under the curve, constructed with a receiver operator characteristic analysis calculated by the *pROC* package, will be used to evaluate individual classifier models as well as the ensemble model’s performance in distinguishing between cancer and noncancer conditions. Clinical diagnosis results, including but not limited to gastroscopy or pathological determinations, are considered the “gold standard.” The cancer risk threshold (cutoff value) will be determined based on the training data set. After that, the prediction performance of the established model will be evaluated in the testing data set in several aspects, including sensitivity, specificity, positive prediction value, and negative prediction value. These variables, as well as 95% CI, will be calculated using the *epiR* package. The Cohen κ score will be calculated with the *vcd* R package to demonstrate the concordance of the predicted results versus the pathological diagnostic results. A single-blind analysis will be applied, which means that the bioinformatics analysts will be blinded to whether the subjects have GC or not. The results are considered significant if the *P* value is <.05. All statistical analyses will be performed in R (version 3.6.3; R Core Team).

### Patient and Public Involvement Statement

Patients, healthy subjects, and the public will not be involved in our studies for reporting, designing, or implementation. We have no intention of sharing this study’s results with the participants, unless they proactively request to receive this information.

### Ethics Approval

The design and implementation of this study strictly followed the Declaration of Helsinki and Chinese medical research norms and regulations. This protocol was approved by the Medical Ethics Committee of the First Affiliated Hospital of the Air Force Medical University (Xi’an, China; approval No. KY20222222-F-1). This study was also registered at ClinicalTrial.gov (NCT05668910).

All enrolled subjects signed informed consent forms for blood sample retention, and they were fully informed that their blood samples would be used for scientific experimental research. A primary principle of this study is to protect the rights and interests of the subjects while ensuring the scientific nature of this study and the authenticity of the data. The subjects shall be volunteering to participate in this study, and their privacy will be fully respected. Throughout this study, all the data will be anonymized and maintained with confidentiality.

## Results

Recruitment began in November 2022 and as of August 2023, we enrolled 250 patients. The last recruitment is expected to finish by April 2024. As for data analysis, it is anticipated to be finalized by September 2024.

## Discussion

The primary aim of this study was to evaluate the effectiveness of using combined cfDNA features, determined through WMS analysis, for early cancer detection. If our model is incorporated into current clinical practices, an individual with a positive result would subsequently undergo either a gastroscopy, a biopsy, or both to ascertain the presence of GC. Conversely, a negative result would indicate a low likelihood of GC and prompt recommendations for a deferred gastroscopy, along with the possibility of an annual repeated test.

The major strength of this study is that this is the first registered observational study, to our knowledge, designed to evaluate the integrated analysis of methylation patterns, fragmentation profiles, and chromosomal copy number changes in cfDNA for early GC detection. Additionally, cfDNA studies designed for the Chinese GC population are limited. Our study will reveal whether multidimensional analysis of cfDNA is an effective strategy for differentiating patients with GC from nonmalignant individuals in the Chinese population.

This study has several potential limitations. Foremost among these is the fact that it is a case-controlled study with a relatively modest cohort size. We acknowledge that case-control studies, especially those performed at a single site, are susceptible to selection bias. Individuals enrolled from a single hospital may not be fully representative of the diversity of a general screening population. Given these limitations, a comprehensive and sizable prospective validation within a screening population will be pursued prior to considering clinical implementation. Another limitation would be that it is impossible to include all the possible benign gastric disease types in the control group; therefore, the constructed model might have limited ability to differentiate GC from other types of benign diseases beyond this study. In addition, although the individuals will be prospectively enrolled in this study, the analysis will be carried out retrospectively. However, it is notable that the bioinformatics analysts will remain blinded to the true pathological outcomes during the validation of the model in the testing cohort, thereby mitigating the potential analysis bias.

Should the results of this research align with our expectations, they will furnish support for the use of ctDNA in early detection of patients with GC. The THEMIS model could significantly contribute by complementing the standard-of-care screening and aiding in the differentiation of high-risk patients warranting further diagnostic evaluation. The findings of this study, looking ahead, could substantially enhance early screening and early diagnosis practices, potentially resulting in a shift toward earlier-stage diagnoses and consequently elevating the 5-year survival rate for patients with GC.

## Abbreviations

CA: cancer antigen
CAFF: chromosomal aneuploidy of featured fragments
cfDNA: cell-free DNA
ctDNA: circulating tumor DNA
FEM: fragment end motif
FSI: fragment size index
G-17: gastrin-17
GC: gastric cancer
IRC: institutional review committee
MFR: methylated fragment ratio
PG: pepsinogen
THEMIS: Thorough Epigenetic Marker Integration Solution
WMS: whole methylome sequencing

## Data Availability

All data generated or analyzed throughout the course of this study, if not already included within this paper or its Multimedia Appendix files, can be obtained from the corresponding author upon submitting a reasonable request for research purposes.
